# Validation and Estimation of Obesity-Induced Intervertebral Disc Degeneration through Subject-Specific Finite Element Modelling of Functional Spinal Units

**DOI:** 10.3390/bioengineering11040344

**Published:** 2024-03-31

**Authors:** Nitesh Kumar Singh, Nishant K. Singh, Rati Verma, Ashish D. Diwan

**Affiliations:** 1 Computational Biomechanics Lab, Department of Biomedical Engineering, National Institute of Technology, Raipur 492010, India; ernksingh24@gmail.com; 2 Biomechanics Lab, School of Biomedical Engineering, Indian Institute of Technology (BHU), Varanasi 221005, India; rati.verma14@gmail.com; 3 Spine Labs & Spine Service, St George & Sutherland Campus, Clinical School of Faculty of Health & Medicine, University of New South Wales, Sydney, NSW 2502, Australia; a.diwan@unsw.edu.au

**Keywords:** degeneration, obesity, lumbar spine, body mass index, intervertebral disc, finite element analysis

## Abstract

(1) Background: Intervertebral disc degeneration has been linked to obesity; its potential mechanical effects on the intervertebral disc remain unknown. This study aimed to develop and validate a patient-specific model of L3–L4 vertebrae and then use the model to estimate the impact of increasing body weight on disc degeneration. (2) Methods: A three-dimensional model of the functional spinal unit of L3–L4 vertebrae and its components were developed and validated. Validation was achieved by comparing the range of motions (RoM) and intradiscal pressures with the previous literature. Subsequently, the validated model was loaded according to the body mass index and estimated stress, deformation, and RoM to assess disc degeneration. (3) Results: During validation, L3–L4 RoM and intradiscal pressures: flexion 5.17° and 1.04 MPa, extension 1.54° and 0.22 MPa, lateral bending 3.36° and 0.54 MPa, axial rotation 1.14° and 0.52 MPa, respectively. When investigating the impact of weight on disc degeneration, escalating from normal weight to obesity reveals an increased RoM, by 3.44% during flexion, 22.7% during extension, 29.71% during lateral bending, and 33.2% during axial rotation, respectively. Also, stress and disc deformation elevated with increasing weight across all RoM. (4) Conclusions: The predicted mechanical responses of the developed model closely matched the validation dataset. The validated model predicts disc degeneration under increased weight and could lay the foundation for future recommendations aimed at identifying predictors of lower back pain due to disc degeneration.

## 1. Introduction

The functional spinal unit (FSU) is the smallest component of the spine, and its biomechanical properties are representative of the rest of the spine’s motion segments. Morphological changes in FSU, including variations in material or structural properties, are key factors that are responsible for progressive irreversible intervertebral disc degeneration (IVDD) [1]. This involves major trauma, destruction of tissues over time, or minor repetitive loading, which reduces the height of the disc and affects the mechanical function of the FSU. Other factors associated with disc degeneration are cell nutrition and transport, mechanical loads, obesity, intense vibration, genetics, and smoking [2,3].

Finite element (FE) modelling is vital for understanding FSU biomechanics under external forces [4]. Critical understandings of FSU responses to treatments come from clinical studies, experiments, and animal models. FE analysis offers cost-effective efficiency, capturing intricate biomechanics that are hard to measure experimentally [5,6]. Developing subject-specific spine models is time-consuming; researchers often opt for linearized models [7]. In view of modelling nonlinear spine behavior, it is noteworthy that spinal FE models frequently undergo validation solely through assessing their end-point responses to specific loads or moments. Thus, rigorous validation ensures accuracy and reliability before numerical exploration [8]. This study initially aims to improve confidence by developing and validating a subject-specific L3–L4 model, enhancing the credibility of FE predictions.

The L3–L4 lumbar vertebrae were chosen for this investigation because of their clinical importance and relevance to the field of spinal biomechanics. Due to its frequent involvement in degenerative disorders such as disc herniation and spinal stenosis, the L3–L4 segment is frequently selected as a therapeutically relevant region for research [9]. Moreover, this level offers a stable and consistent anatomical location for biomechanical study because it is located in the middle of the lumbar spine [10]. The L3–L4 level is also easily accessible and visualized, which makes it a good choice for study, especially when imaging or surgical procedures are involved [11]. This study assures consistency with existing literature and facilitates comparisons with prior findings by specifically focusing on the L3–L4 vertebrae [12].

Obesity is a global health issue that is gradually becoming worse [13,14]. In 2019–21, 22.9% of men and 24% of women in the age group 15–49 were obese or overweight [15,16]. To identify the persons as obese or overweight, the body mass index (BMI) classification is widely adopted, as given in Table 1 [17,18].

Obesity and overweight increase the load on the lumbar spine, potentially contributing to IVDD, which is the prominent reason for lower back pain (LBP) [19,20,21,22]. The high BMI reduces the height of the vertebral disc as the amount of gelatinous mass in the nucleus pulposus (NP) is reduced. As a result, the hydrostatic pressure rises and the outer annulus begins to swell. Also, an increase in BMI increases the stresses on the vertebrae when the human body is in diversified motions [23,24].

To examine LBP in obesity, a potential approach involves assessing the impact of reduced body weight on back pain, essentially evaluating whether weight loss alleviates the condition. In a systematic review evaluating the impact of BMI reduction following weight loss surgery, we have demonstrated [25] that in the severely obese, there is a benefit on back pain when weight is lost following surgery.

IVDD is influenced by various factors, such as range of motion (RoM), deformation, and stress distribution within the FSU. Recent research indicates that variations in BMI can affect the morphology and function of IVD over time; however, the link between BMI and IVD degeneration is complex [26,27]. The initial phases of IVDD may exhibit increased segmental mobility or hypermobility, which could result in biomechanical instability. Moreover, biomechanical research has shown that higher BMI leads to elevated von Mises stress (VMS) and deformation in the intervertebral disc (IVD), potentially contributing to degenerative alterations [26,27,28,29,30]. Although these findings provide valuable information, the practical importance of these biomechanical factors in relation to intervertebral disc (IVD) health and pathology is still being studied. Further research is needed to understand their impact on spinal function and degenerative conditions.

To better understand the impact of body mass on IVD mechanics with a view to personalizing treatment strategies, it may be helpful to elucidate mechanical properties of the disc in persons of varying body weight. A in silico model utilizing FE analysis may have a role.

The objective of this study was two-fold. Firstly, to develop and validate the patient specific FE model of the FSU. Secondly, to analyze the effects of increased BMI on the validated FSU to investigate the IVDD during compressive loading following extension, flexion, lateral bending and axial rotation. Stress, RoM, and deformation were assessed to estimate IVDD.

## 2. Materials and Methods

**Figure 1:**
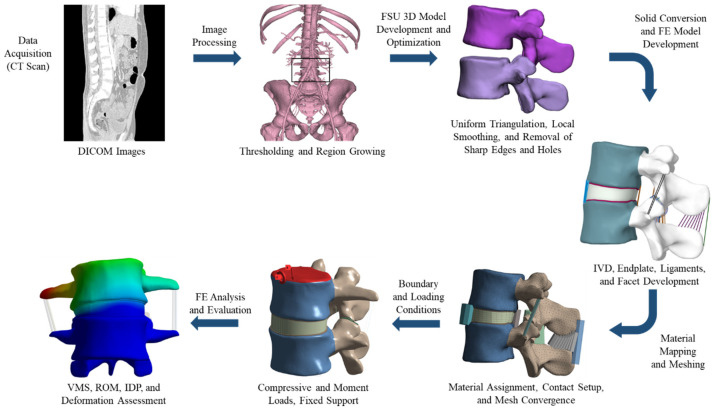
Workflow of the FSU study. (The reader is directed to the online version of this article for interpretation of the references to colour in this figure legend).

A three-dimensional (3D) model of FSU components was developed, followed by model optimization to make it suitable for FE analysis, which was later assembled on the patients geometrical coordinate system. Numerical simulation was subsequently performed and validated the model with previous literature reports. The complete steps involved in this work are summarized in Figure 1. Finally, the validated model was used to assess the impact of increased weight on IVD.

### 2.1. FE Model Development of FSU

The advancement of high-performance computing and sophisticated computer simulation techniques has stimulated the creation of 3D computational anthropomorphic models representing the anatomy and physiological functions of the human spine. Various methodologies have been employed to develop these 3D computational models, with popular approaches including consistency-based volumetric rendering techniques such as maximum intensity projection, curved planar reformation, ray tracing, and shaded surface display [31,32,33,34]. Direct volume rendering techniques such as ray casting, spatting, shear warp, and texture mapping have also been reported. Additionally, regularization-based surface reconstruction methods, including point spread function reconstruction, convolutional network 3D reconstruction, and hierarchical deformable model-based reconstruction, have been used in several studies [35,36,37,38,39].

Current 3D reconstruction algorithms are integrated into modern 3D medical imaging platforms such as Syngo, 3D Slicer, Amira, and Mimics [40]. These platforms use magnetic resonance imaging (MRI) or computed tomography (CT) images to generate 3D models, enabling more personalized analysis of spinal conditions.

In this study, the Materialise MIMICS 18.0 (Materialise, Leuven, Belgium) platform was employed to develop the bony component of the functional spinal unit (FSU) model. The CT images of a lumbar spine were obtained from a healthy 27-year-old male in Digital Imaging and Communications in Medicine (DICOM) format. The CT scan DICOM images were imported into the MIMICS and processed with a semi-automatic region growing algorithm to obtain a 3D model of L3–L4 [41]. The 3D model of the L3–L4 was optimized and exported as a STEP file and imported into ANSYS SpaceClaim v19.0R2 (Canonsburg, PA, USA). Cortical and cancellous bones were separated from the vertebral body, while posterior bone was left intact. Cortical bone thickness was established by giving an internal offset of 0.5 mm [42]. Material properties of bone were represented as an isotropic, linear material with an elastic modulus that depends on the bone mineral density (BMD) as determined by the CT scans [43]. Hounsfield units were scaled to g/cm^3^ using a scale factor, and these values were regarded as equivalent to the BMD equivalent values ${(\rho }_{QCT})$. Equation (1) was used to determine the apparent density ${(\rho }_{App})$ from the ${(\rho }_{QCT})$ [44]. In Equations (1) and (2), E is expressed in MPa and ρ in g/cm^3^.

(1) $$\rho_{QCT}=\rho_{App}\times 0.6\left(\frac{g}{{cm}^{3}}\right)$$

The apparent density and modulus equation tailored for vertebrae was used to obtain Young’s modulus Equation (2) [45].

(2) $$E_{bone}=4730{\;\rho }_{App}^{1.56}\;MPa$$

**Figure 2:**
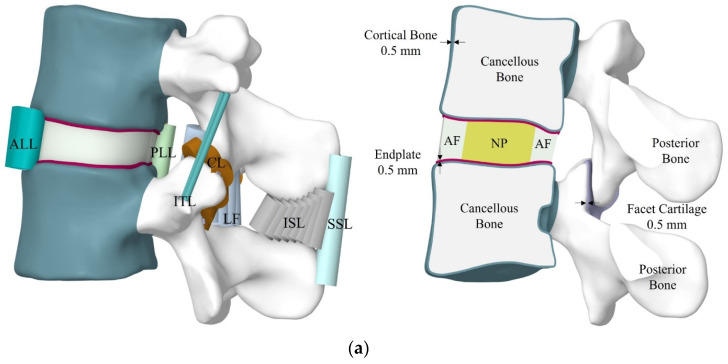

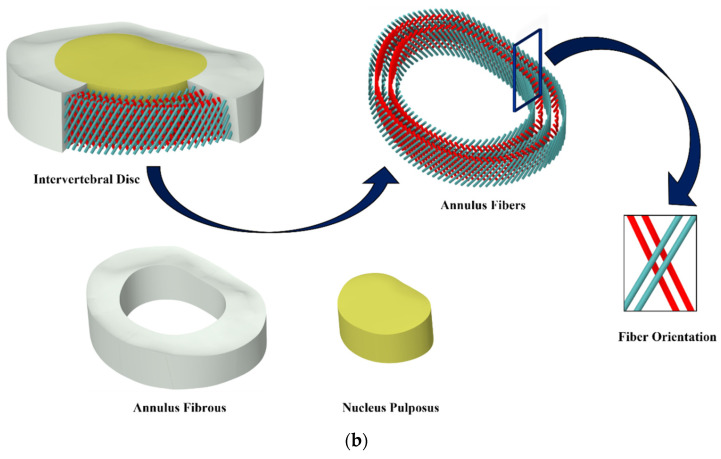
FE model of the (**a**) FSU and their sectional view with associated thickness of components and (**b**) IVD with fibers.

The thickness of vertebral endplates on the inferior and superior surfaces of the vertebral bone was taken as 0.5 mm [46,47,48]. The articular facets were modelled with a thickness of 0.5 mm, whereas the initial gap between the two articular facets was considered 0.5 mm to transmit compressive force only [49,50,51]. A frictional contact was used to make contact between the facets with a friction coefficient of 0.1, and bonded contact was used for the remaining FSU joints [52]. The seven ligaments used in the model were developed by tension-only node-to-node truss elements [53], namely posterior longitudinal ligament (PLL), capsular ligament (CL), anterior longitudinal ligament (ALL), ligamentum flavum (LF), intertransverse ligament (ITL), supraspinous ligament (SSL) and interspinous ligament (ISL). The standard cross-sectional area and material properties of each ligament were adopted from the previous literature. Figure 2a illustrate the FSU and their sectional view with associated thickness of components.

The 3D model of the IVD was separated into annulus fibrosus (AF) and NP, where the AF covered almost 60% of the total volume of the IVD [42,54,55]. The AF was modelled as a composite reinforced by collagen fibers in concentric rings [51]. Two evenly spaced layers of fibers with an orientation of roughly ±30° to the horizontal plane made up the five layers of lamellae that encircled the NP in a crisscross-like manner [56,57]. Figure 2b represents the IVD with fiber orientation. The diameter of the fibers was 0.5 mm and the distance between each fiber was taken to be 0.75 mm [57]. Only tension-resisting node-to-node truss elements were used to define the fibers [58]. The truss elements cross-sectional area was calculated as per the standard relation reported by Goel et al., 1995 [57]. The Mooney Rivlin constitutive model was used to model the nonlinear NP and AF ground substance characteristic as incompressible and hyperelastic material [1,59]. A two-parameter Mooney-Rivlin formulation is expressed in Equation (3) [58,60].

(3) $$W=c_{01}\left(I_{2}-3\right)+c_{10}\left(I_{1}-3\right)+\frac{1}{d}\;{\left(J-1\right)}^{2}$$

where:
*W*Strain Energy Function*I*_1_ and *I*_2_First/second deviatoric strain invariants*c*_01_ and *c*_10_Material constants*d*Incompressibility parameter of the material*J*Elastic volume ratio

All components of FSU except the NP and AF ground substance have been modelled with linear isotropic material properties [43]. Table 2 summarizes the material properties of each FSU component.

### 2.2. Boundary and Loading Conditions

**Figure 3:**
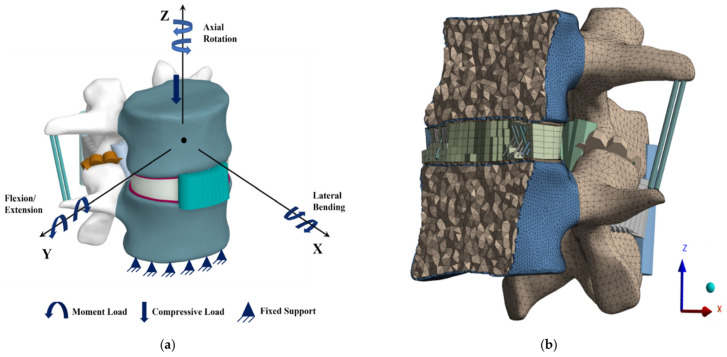
Illustration of meshing and boundary conditions of L3–L4 FSU. (**a**) loading and boundary conditions; (**b**) internal mesh model.

The combined load (compressive load and moment load) was applied on the upper surface of the L3 vertebral body to simulate a RoM in all directions, while the lower part of the L4 vertebra was completely immobile. Figure 3a shows the boundary and physiological loading conditions on FSU. The loading environment in this work involved two separate sets.

First, the FE model of FSU was loaded for the validation purpose using combined load listed in Table 3 [67,68,69]. These compressive forces and moments were derived from previously investigated FE models that accurately represented the maximum RoM. The intradiscal pressures (IDP) and RoM of the FE model were compared with earlier well-established FE investigations [70]. In lateral bending and axial rotation, the average of right and left was chosen for FE model comparison [71].

Second, the validated model was used to estimate the IVDD in accordance with BMI variability. Based on average weight and height, the BMI was calculated from data released in 2020 by the Indian Council of Medical Research (ICMR) for ages ranging from 19–39 years [72]. It is important to highlight that, although the Indian population were chosen as a representative sample data, obesity BMI criteria are the same worldwide, hence the results can be readily globalized. BMI was calculated and grouped into four categories that have shown a substantial increase from the standard index (Table 1). Therefore, the new BMI $\left({BMI}_{N}\right)$ for each group is initially partitioned into two parts Equation (4).

(4) $${BMI}_{N}={BMI}_{S}+{BMI}_{I}$$

where ${BMI}_{S}$ is the standard BMI for each group and ${BMI}_{I}$ = 3.28 kg/m^2^ is the increased BMI calculated from the available data of ICMR. So, the ${BMI}_{N}$ for each group is listed as: I (underweight) < 18.5 kg/m^2^, not included in the study as data for this group was not available in records of ICMR; II (normal weight) = 21.78 kg/m^2^; III (overweight) = 28.28 kg/m^2^); IV (obese) = 33.28 kg/m^2^. Based on the calculated ${BMI}_{N}$, the corresponding load for the last three groups was calculated: Group II: 637 N, Group III: 826.37 N, and Group IV: 972.94 N. The FE investigations were undertaken on standard boundary conditions used for validation, except the compressive load was changed based on the ${BMI}_{N}$ calculated for the last three groups. However, only a lower range of BMI in each group was used to estimate load, because it is sufficient to show the effect of load on BMI changes among each categorized group.

### 2.3. Mesh Convergence

A mesh convergence study was performed to determine the ideal mesh size, and meshing was performed in Ansys (ANSYS Inc., Canonsburg, PA, USA). Three meshes were generated sequentially: Mesh A (1.5 mm), Mesh B (2 mm), and Mesh C (2.5 mm) [73]. A 500 N compressive force was applied on the top of the L3 vertebra. The VMS of FSU components was estimated and compared in the FE model. When the results of two consecutive mesh resolutions differed less than 5%, the mesh was considered to be convergent [74,75]. The mesh model of the FSU is depicted in Figure 3b.

**Figure 4:**
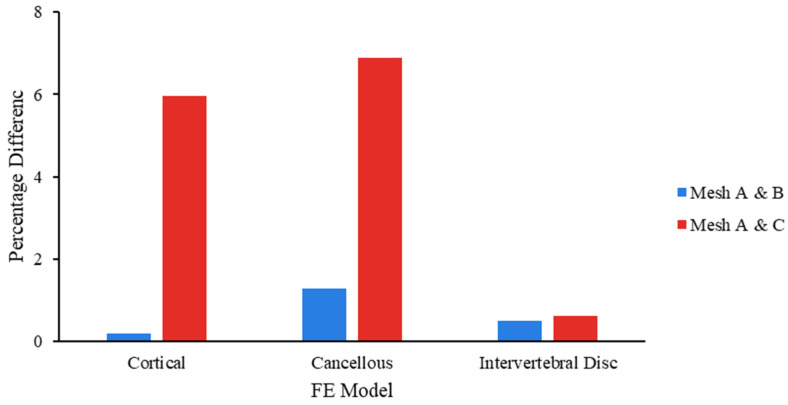
Percentage variation in VMS between Mesh A and B model, and between Mesh A and C model, subject to axial compression.

In terms of VMS, the highest percentage difference was observed between Mesh A and C, which was 6.89% in the cancellous component, whereas a difference of 0.5% was observed between Mesh A and B, while Mesh B and C differed by 0.62% in the IVD component. All components of the model have less than 5% VMS variations between mesh A and B; however, variation between mesh A and C exceeded the 5% threshold in components. Figure 4 shows the percentage variation in VMS between Mesh A and B, as well as between Mesh A and C for cortical, cancellous, and IVD. Thus, Mesh B was taken into consideration, and the entire FSU was meshed with tetrahedral elements of 2 mm edge length, though IVD was meshed with hexahedral elements to obtain an optimal FE model [76]. The meshed FE model of FSU consists of approximately 273,493 elements and 26,936 nodes, and the individual component elements and nodes are listed in Table 2. The run-time for each set of the loads range was 1–2 h (the PC used in this study was FusionStor with Intel^®^ Xeon^®^ Gold 5218 CPU @ 2.30 GHz processor, Nvidia Quadro RTX 5000 GPU and 64 GB RAM).

## 3. Results

### 3.1. Validation of FSU-FE Model

**Figure 5:**
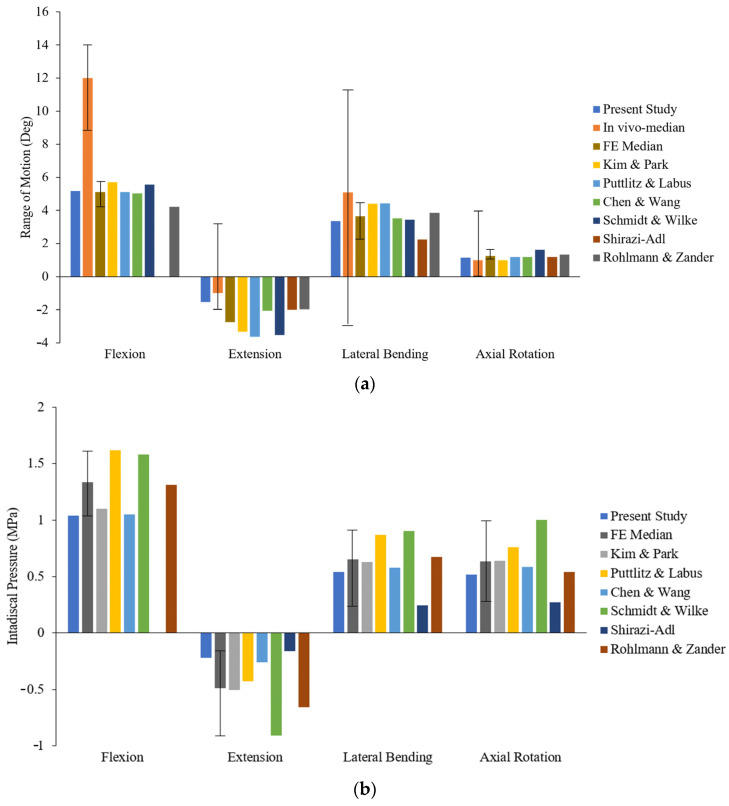
Comparison between predicted results with previous studies (**a**) RoM of L3–L4 (**b**) IDP of IVD.

All previously reported FSU numerical models, however, are dependent on a wide range of input parameters with a considerable amount of uncertainty. As a result, the key method for assessing the FE model reliability and accuracy in relation to its application context is validation. In the last two decades, several papers have been published in the field of biomechanics, highlighting the significance of validation and offering guidance for these approaches [77,78,79]. As a consequence, RoM and IDP at the L3–L4 level were compared with well-established FE and in vivo studies of the lumbar spine to validate our FE model. For the developed FSU, RoM was 5.17° in flexion, 1.54° in extension, 3.36° in lateral bending, and 1.14° in axial rotation. The present study was compared with previous in vivo studies [80,81,82,83], then it was compared with a mean of all FE studies, as well as the results of six other FE studies [69,74,84,85,86,87], as combinedly reported by Dreischarf et al., 2014 [70,88]. It must be highlighted here that, despite the slight variation in RoM from the earlier published data, the median of all the data was taken for comparison of our results. We hypothesized that the median of the data would give a closer comparison of results instead of using individual data. The RoM predicted on a similar set of loading and boundary conditions across L3–L4 segments showed an excellent agreement with those reported in the literature (Figure 5a).

Furthermore, IDP of IVD located at the midof the L3–L4 spinal segment was assessed for all postures. The FE simulation results of IDP were 1.04 MPa in flexion, 0.22 MPa in extension, 0.54 MPa in lateral bending, and 0.52 MPa in axial rotation, respectively. For IDP under all loading conditions, the current FE results are in good agreement with the FE median reported by Dreischarf et al., 2014 [70] and six individual FE studies [69,74,84,85,86,87], as shown in Figure 5b. Overall, the predicted FE results of RoM and IDP in this study satisfactorily agreed with different studies for different postures. Therefore, the developed FE model could generate adequate and optimized results, which were subsequently used to estimate the effect of increased BMI on the degeneration of IVD.

### 3.2. Effect of BMI on FSU

**Figure 6:**
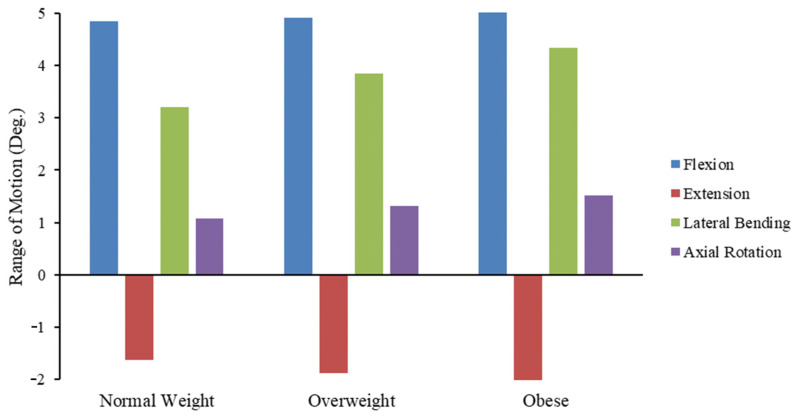
Comparison of RoM between normal weight, overweight, and obese in all degrees of freedom.

As per the BMI estimated in Section 2.2, the corresponding compressive load calculated were Group II (normal weight): 637 N, Group III (Overweight): 826.73 N, and Group IV (Obese): 972.94 N, respectively. Under these compressive loads and the set of moment loads for different postures mentioned in Table 3, the RoM of the L3–L4 segment, VMS, and deformations of IVD were, respectively, estimated. The RoM of normal weight and overweight was 4.85° and 4.92° in flexion, 1.64° and 1.88° in extension, 3.21° and 3.84° in lateral bending, and 1.08° and 1.31° in axial rotation, respectively. The RoM for the obese classification was estimated as 5.02° in flexion, 2.06° in extension, 4.33° in lateral bending, and 1.51° in axial rotation. Figure 6 shows the comparison of RoM between normal weight, overweight, and obese in all postural conditions.

**Figure 7:**
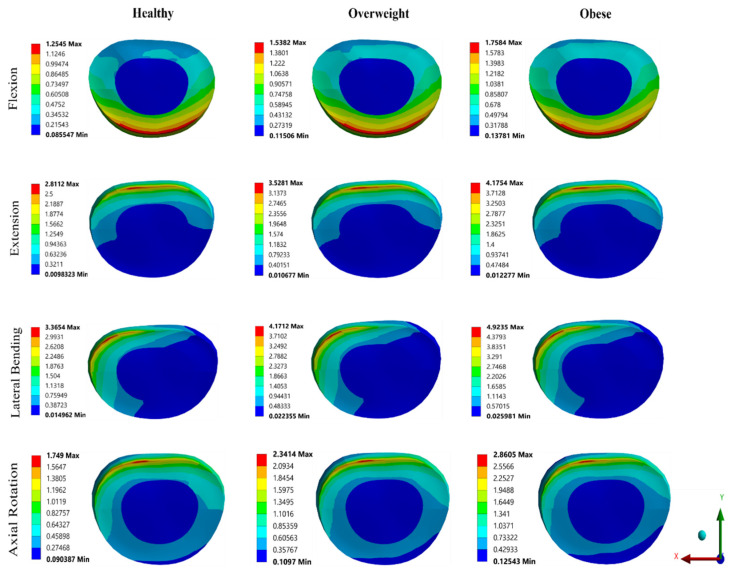
VMS contours on IVD.

**Figure 8:**
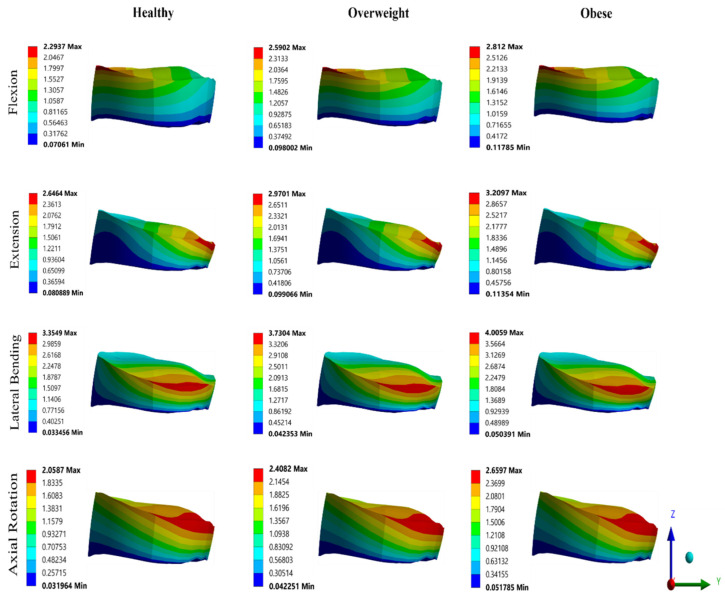
Deformation contours on IVD.

Furthermore, VMS and deformation contours of the IVD in all three categorized groups for different moments are depicted in Figure 7 and Figure 8. There is clear evidence of the effect of increased weight; as the weight increases, the stress distribution and deformation on the IVD increases. The maximum stress on the IVD was 4.92 MPa in lateral bending for obese, and the minimum stress was 1.25 MPa in flexion for normal weight. From Figure 7, it can be observed that there is almost a linear increase in stresses when the corresponding weight of the subject was increased, i.e., normal weight to obese: the IVD stress increased from 1.25 to 1.76 MPa in flexion, 2.81 to 4.18 MPa in extension, 3.36 to 4.92 MPa in lateral bending, and 1.75 to 2.86 MPa in axial rotation. It can be observed that as BMI increases, there is an almost linear increase in RoM. Similar trends were found in total deformation plot; as the weight increased, the deformation on the IVD increases. Figure 8 shows the deformation of the IVD in normal weight, overweight, and obese in all different directions. When the load was increased according to the corresponding classified group, the IVD deformation increased from 2.29 mm to 2.81 mm in flexion, 2.65 mm to 3.21 mm in extension, 3.35 mm to 4.0 mm in lateral bending, and 2.06 mm to 2.67 mm in axial rotation respectively. It was observed that the RoM of the L3–L4 segment, VMS, and total deformation of IVD were maximum for obesity, followed by overweight and normal weight.

## 4. Discussion

Validating specific FE models for the L3–L4 lumbar vertebrae and conducting preliminary investigations on IVDD due to increased BMI is essential to understand the biomechanical mechanisms underlying spinal health and pathology. By validating these models, researchers ensure the accuracy of the computational simulations used to predict the effect of different loading conditions on the spine. Investigating the effects of increased BMI on IVDD may provide valuable insight into the role of mechanical loading in disc health and disease progression, informing preventive strategies and therapeutic interventions aimed at reducing the burden of spinal disorders. Overall, these research efforts contribute to advancing our understanding of spinal biomechanics, facilitating the development of targeted interventions to reduce the impact of degenerative disc diseases, development and effect of new implant, and improve spinal health outcomes [89].

A 3D patient-specific FE model of healthy FSU was developed by using CT scan DICOM images, including a detailed realistic description of the annulus and nucleus geometry, and incorporated 3D spar elements listed in Table 2 to simulate the ligaments pragmatically. The model was validated by comparing the results from existing in vivo and FE literature. Further, the validated FSU model was used to investigate the effect of BMI variation on IVDD for the Indian cohort.

A mesh convergence study has been performed, and the suitability of mesh was assessed based on VMS criteria [83]. The three FE models of L3–L4, consisting of A, B, and C models, were used to study the mesh convergence. In contrast to models B and C, which showed 5.96% deviation in cortical bone, 6.89% deviation in cancellous bone, and 0.62% deviation in IVD, whereas models A and B differed by 0.18% in cortical bone, 1.28% in cancellous bone, and 0.5% in IVD. So, models A and B showed below 5% difference in the VMS parameter in all the components. Thus, model B was used for FSU with an optimal mesh size of 2 mm.

The initial phase of this study involved the validation of the lumbar spine FE model. As shown in Figure 6, the RoM in this investigation followed a similar pattern as in the existing in vivo and FE estimations. During flexion, the RoM was within the range of FE the median, whereas a lower range was observed while comparing the result with in vivo median. However, when compared with individual six FE studies, obtained flexion values were greater compared to those published by Rohlmann and Zander et al., 2009 [69]; lower by Kim and Park et al., 2013 [84], and Schmidt and Wilke et al., 2012 [86]; and within the limits of Putlitz and Lebus et al., 2011 [74], and Chen and Wang et al., 2001 [85]. The RoM during the extension was significantly smaller than the median results reported in the previous six FE models. In the case of lateral bending, the result was within the range of Chen and Wang et al., 2001 [85], Schmidt and Wilke et al., 2012 [86], and Rohlmann and Zander et al., 2009 [69]; higher than Shizazi-Adl et al., 1994b [87], and lower than Kim and Park et al., 2013 [84], and Putlitz and Lebus et al., 2011 [74]. The study by Shizazi-Adl et al., 1994b [87], did not include the flexion motion of the RoM and IDP. The RoM during axial rotation was similar to the FE median and in vivo median values from previous studies.

From Figure 5b, the IDP results of this study showed a similar pattern with the IDP shown in the FE median. However, comparing the IDP with individual FE studies, the IDP value published by Putlitz and Lebus et al., 2011 [74], and Schmidt and Wilke et al., 2012 [86], was greater, whereas the IDP reported by Shizazi-Adl et al., 1994b [87], was slightly lower than the present study in all postural conditions. Furthermore, the values reported by Rohlmann and Zander et al., 2009 [69], were also large in all motions except in axial rotation. Additionally, IDP values during the flexion, axial rotation, and lateral bending motions were found to be in range compared to the study published by Kim and Park et al., 2013 [84], and Chen and Wang et al., 2001 [85]. It can be clearly observed from Figure 5b that Kim and Park et al., 2013 [84], reported larger values, whereas values reported by Chen and Wang et al., 2001 [85], were in the range during the extension motion of the L3–L4 segment.

Some of the published data sets showed discrepancies. For instance, investigations on extension, flexion, axial rotation, and lateral bending show similar results, although the extension results were quite inconsistent. The disparity between the data sets was believed to be caused by the difference in subject age, a moderate degree of orientation change during subject scanning, fixation, and the resulting boundary conditions. Moreover, the contact between two facet joints will increase the stiffness of the FSU segment in extension since variations in the initial orientation at the facet joint will also have a substantial impact [90].

In the last stage of this study, the validated FSU model was used to illustrate its capabilities and effectiveness in numerical analysis. To demonstrate this, the influence of higher BMI on IVDD was estimated. BMI under different categorized groups was used to investigate the IVD during extension, flexion, axial rotation, and lateral bending motions. BMI appeared to influence the RoM, distribution of VMS, and deformation in the lumbar spine, which was already discussed in Section 3.2 in detail.

The transition from each BMI group (II, III, IV) for the FE model represented a change in body mass of 65 kg, 84.36 kg, and 99.28 kg, respectively, which were calculated from ICMR data. The two consecutive BMI levels were compared with the reference point of Group II (Normal Weight BMI: 21.78 kg/m^2^) BMI results [91]. The obtained results from the FE simulations reflect the overweight classification (Group: III) with a BMI of 28.28 kg/m^2^, who gains 19.36 kg of body weight will increase RoM of L3–L4 spine segment by 1.43% during flexion, 13.64% during extension, 17.84% during lateral bending and 19.25% during axial rotation. Furthermore, VMS and deformation in the L3–L4 spine segments also increased by 20.79% and 12.29%, 22.71% and 11.39%, 21.51% and 10.73%, and 28.85% and 15.66%, respectively, during extension, flexion, axial rotation, and lateral bending.

Similarly, simulation results for the obese population are more significant, as the Indian population with an average BMI of 33.28 kg/m^2^ who gains 34.28 kg of body weight from the normal weight will see an increase in the RoM of 3.44% in flexion, 22.07% in extension, 29.7% in lateral bending and 33.2% axial rotation. VMS and deformation both showed a similar tendency as RoM increased the values also increased. During extension, flexion, axial rotation, and lateral bending, the VMS increased by 33.89%, 39.2%, 37.68%, and 48.16%, while the deformation increased by 20.39%, 19.11%, 17.69%, and 25.42%, respectively.

From the RoM shown in Figure 6, it is possible that increased RoM in different posture may increase the stress on the AF and stimulate the degeneration of IVD, mainly the NP [92]. This may trigger pain and rip the AF at the disc [93,94].

These result such as RoM, stress and deformation potentially provide a vital understanding of the risk of IVDD of the lumbar spine and the effect of moderate weight control on patient spine health, as studies have shown a link between disc degeneration incidence rate and increase in the BMI. Overall, the findings suggest a potential association between BMI and IVDD, but it is crucial to acknowledge that this relationship is observational in nature, implying that it is based on observed pattern of increased biomechanical parameters. Therefore, care should be taken when inferring direct correlations between BMI and IVDD. Numerous studies highlight the considerable difficulties confronted by spine surgeons and researchers when identifying reliable predictors of LBP using static clinical images. This outcome may serve as a basis for forthcoming recommendations focused on identifying predictors for LBP caused by disc degeneration [95,96,97,98,99,100].

It is important to mention the shortcomings and oversimplifications of the current FE investigation, of which we are well aware. The models that have been presented have the potential to be improved upon, taking into account the effects of muscle forces, variations in the size and location of ligaments and fibers, computer-simulated IVD, facet cartilage, and endplate constructed by MRI data, as well as including material properties of the subject-specific FSU components used in this study [101]. Other limitations include the level of degeneration and factors such as cell nutrition and transport, intense vibration, genetics and smoking that affect the IVD, were not addressed in this investigation [2,3]. By addressing these simplifications, the model could be improved, but we expect the relationship between the FE model and their results would remain the same. As a result, we believe that the validated model is appropriate for further spine-related biomechanical research objectives [102]. However, despite the use of sophisticated computational models and their in-depth analyses, it is essential to acknowledge the inherent uncertainties present in such studies. The uncertainties in this FSU study can arise from several factors, such as the fact that development of a patient-specific FE model may not exactly mimic the biomechanical behavior of the FSU [103]. Furthermore, the gap between superior and inferior facet cartilage was varied, as this parameter cannot be easily determined from CT scans [104]. Variability in assigning material properties to biological tissues is challenging due to their high nonlinearity, heterogeneity and time-dependent nature [4]. The boundary conditions applied in computational models may not precisely mimic the exact complex physiological loading conditions and differences between in vitro and in vivo conditions can also contribute to uncertainties in FSU model [105,106]. The inherent variations in anatomical characteristics among the patients can introduce uncertainties into the outcomes and constrain their reliability [107].

It should be highlighted that this type of validation does not necessarily represent the conditions found in vivo, as the FE model are unable to replicate the real-world scenario. The most considerable initial step is typically validating the FE model against in vivo studies. It is unlikely that the model will be able to represent the more complex scenario in vivo if it cannot accurately represent the controlled conditions in the laboratory. Overall, the results show that this 3D FE model performed well during the estimation of IVDD due to increased BMI, and the model could be used in future spine related research objectives.

## 5. Conclusions

This study included the development and validation of a 3D FE model of the FSU. Mesh sensitivity analysis was conducted to assess the robustness of the FE model, with mesh convergence achieved by evaluating VMS across various FSU components. The validated models analyzed through the comparison of ROM and IDP with both experimental data and simulation outcomes from established FE models, as well as in vivo data adopted from the literature. The validation process covered a combined (compressions and moments) loading modes indicative of real-world lumbar spine movements. Overall, the simulation results closely aligned with experimental data and predicted outcomes from the existing literature, affirming the validity and accuracy of the modelling approach introduced in this study. Investigation for the impact of BMI on spinal biomechanics using the validated FSU model highlights a notable association between increased BMI and heightened stress and deformation in the lower spine, potentially predisposing individuals to disc degeneration. Specifically, the NP emerged as particularly susceptible under these circumstances compared to the AF. Irrespective of spinal position, individuals with elevated BMI exhibited heightened tension on the IVD, suggesting an increased risk of early disc injury. Our study highlights the importance of weight management as a preventative measure for spinal health. Using FE modelling, our findings underscore the reliability and precision of biomechanical predictions pertaining to the FSU. This validated model holds potential for guiding future investigations targeting lumbar spine pathologies, including disc degeneration and scoliosis.

## Figures and Tables

**Table 1:** Distribution of standard BMI in different categories.

BMI Category	BMI (kg/m^2^)
Underweight	<18.5
Normal weight	18.5 to 24.9
Overweight	25 to 29.9
Obesity	≥30

**Table 2:** Material properties of lumbar FE model.

Components	Element Type	Elements	Nodes	Density (g/cm^3^)	Young’s Modulus (MPa)	Poisson’s Ratio	Cross-Sectional Area (mm^2^)	References
**Bone**								
Cortical Bone	Solid 185	33,934	101,066	1.91	12,000	0.3	-	[61,62]
Cancellous Bone	Solid 187	76,347	112,474	1.87	100	0.2	-	[61,62]
Posterior Bone	Solid 187	68,897	109,181	1.87	3500	0.25	-	[63]
**Intervertebral Disc**								
Nucleus Pulposus	Solid 185	965	1290	1.0003	C_10_ = 0.12, C_01_ = 0.09 D_1_ = 1			[64]
Annulus Fibrosus	Solid 185	1440	1960	1.0003	C_10_ = 0.56, C_01_ = 0.14 D_1_ = 1			[64]
**Annulus Fibers**								
Outermost	Link 180	100	400	1.0003	550	0.3	0.196	[65]
Second	Link 180	92	368	1.0003	503	0.3	0.196	[65]
Third	Link 180	85	340	1.0003	455	0.3	0.196	[65]
Fourth	Link 180	79	316	1.0003	408	0.3	0.196	[65]
Fifth	Link 180	71	284	1.0003	360	0.3	0.196	[65]
**Endplate**	Solid 185	6962	19,077	1.0003	23.8	0.4	-	[63]
**Facet Cartilage**	Solid 185	1964	5216	1.0003	35	0.4	-	[66]
**Ligaments**								
Anterior Longitudinal	Link 180	9	36	1.0003	7.8	0.3	63.7	[66]
Posterior Longitudinal	Link 180	5	20	1.0003	10.0	0.3	20	[66]
Ligamentum Flavum	Link 180	11	44	1.0003	15.0	0.3	40	[66]
Inter Transverse	Link 180	4	16	1.0003	10.0	0.3	1.8	[66]
Inter Spinous	Link 180	8	32	1.0003	10.0	0.3	40	[66]
Supra Spinous	Link 180	1	4	1.0003	8.0	0.3	30	[66]
Capsular	Link 180	20	80	1.0003	7.5	0.3	30	[66]

**Table 3:** Combined compressive and moment load on different physiological motion.

Direction of Loading	Compressive Force (N)	Moment (Nm)
Lateral Bending	700	7.8
Axial Rotation	720	5.5
Extension	500	7.5
Flexion	1175	7.5

## Data Availability

The data used in this study is not available for public sharing due to confidentiality and privacy reasons.
